# NIR-spectroscopy for bioprocess monitoring & control

**DOI:** 10.1186/1753-6561-7-S6-P29

**Published:** 2013-12-04

**Authors:** Marko Sandor, Ferdinand Rüdinger, Dörte Solle, Roland Bienert, Christian Grimm, Sven Groß, Thomas Scheper

**Affiliations:** 1 Institut für Technische Chemie, Leibniz Universität Hannover, Callinstraße 5, D-30167 Hannover, Germany; 2 Sartorius Stedim Biotech GmbH, August-Spindler-Straße 11, D-37079 Göttingen, Germany

## Introduction

The Quality by Design (QbD) approach shows significant benefit in classical pharmaceutical industry and is now on the cusp to a stronger influence on biopharmaceutical applications. Monitoring the critical process parameters (CPP) applying process analytical technologies (PAT) during biotechnological cell cultivations is of high importance in order to maintain a high efficiency and quality of a bioprocess. For parameters like glucose concentration, total cell count (TCC) or viability a robust online prediction is in many applications not yet possible. This gap can be closed with the help of NIR spectroscopy (NIRS), which provides quantitative prediction of single analytes in real-time.

For accurate process control based on NIR spectroscopy, special care has to be taken while building the calibration model [1,2]. In cell cultivation almost all analytes are confounded and show large correlation coefficients. Therefore, partial least square (PLS) models are not able to discriminate between the signals of the different analytes. Especially, analytes like glucose or glutamine which are strongly confounded with cell growth need to be evaluated carefully as cell growth is the analyte causing the largest changes in NIR spectra throughout a cultivation run. Spiking experiments are the most efficient way in order to break correlations between critical analytes like glucose and other nutrients or TCC. This strategy should be followed in order to build robust calibration models without correlations [3,4]. Another very critical issue occurring in cell cultivation are batch-to-batch variations. As it is recommended in good modeling practice [5], for robust models it is crucial to use several complete batches for validation which are not part of the calibration set rather than cross validation [6].

## Materials and methods

CHO-K01 cells (Cell Culture Technology, University of Bielefeld), were cultivated in a BIOSTAT^® ^C plus bioreactor (Sartorius Stedim Biotech) with a 7.5 L working volume. In total, eight cultivation runs were performed, each lasting six days on average. Sampling was performed every three to six hours, and reference analytics of the critical process parameters, such as TCC, viability (TC10 automated cell counter, Bio-Rad), glucose, lactate, glutamine, etc. (YSI 2700, YSI Inc.) were determined in the laboratory.

## Results

Table 1 gives an overview of the models and the accuracy of predictions for several analytes investigated. An excellent model could be obtained for total cell count (TCC). Viability can be predicted and glucose can be predicted as well. Correlations from glucose with other analytes have been reduced by spiking of glucose in one cultivation. Predictions for low concentration analytes like glutamine seem to be also predictable at the first glance, but are strongly related to correlations with other parameters, such as TCC. Models based on correlations are not recommended for process control since they show a lack of sensitivity to the analyte of interest and robustness. Whether a model is based on correlations can be easily demonstrated by spiking experiments. Glutamine, for example, was spiked in one cultivation at the end of the batch-phase up to 1 g/L. The glutamine model was not able to predict the spiking, which proves the strong correlation to other analytes. Glutamine cannot be measured directly in this concentration range using NIRS. However, qualitative models on overall nutrient consumption or metabolite accumulation yield promising results (data not shown).

**Table 1:** NIR results for calibration models and validation by external data sets.

Analyte	Range	No. Cal. No. Val. Batches (Samples)		Reg. maths	Factors	SEC	SEP
TCC (·10^6 ^cell/mL)	0-16	5 (185)	3 (118)	None	2	1.07	0.48
Viability (%)	10-100	5 (193)	3 (110)	None	4	4.2	4.2
Glucose (g/L)	0-9	5 (198)	3 (105)	None	4	1.2	0.48
Glutamine (g/L)	0-1.1	5 (189)	3 (114)	SNV	2	0.16	correlation

(TCC: total cell count; No.Cal.: Number of batches (samples) of the calibration set. No.Val.: Number of batches (samples) of the validation set; SNV: standard normal variate; SEC: standard error of calibration; SEP: standard error of prediction)

**Figure 1:**
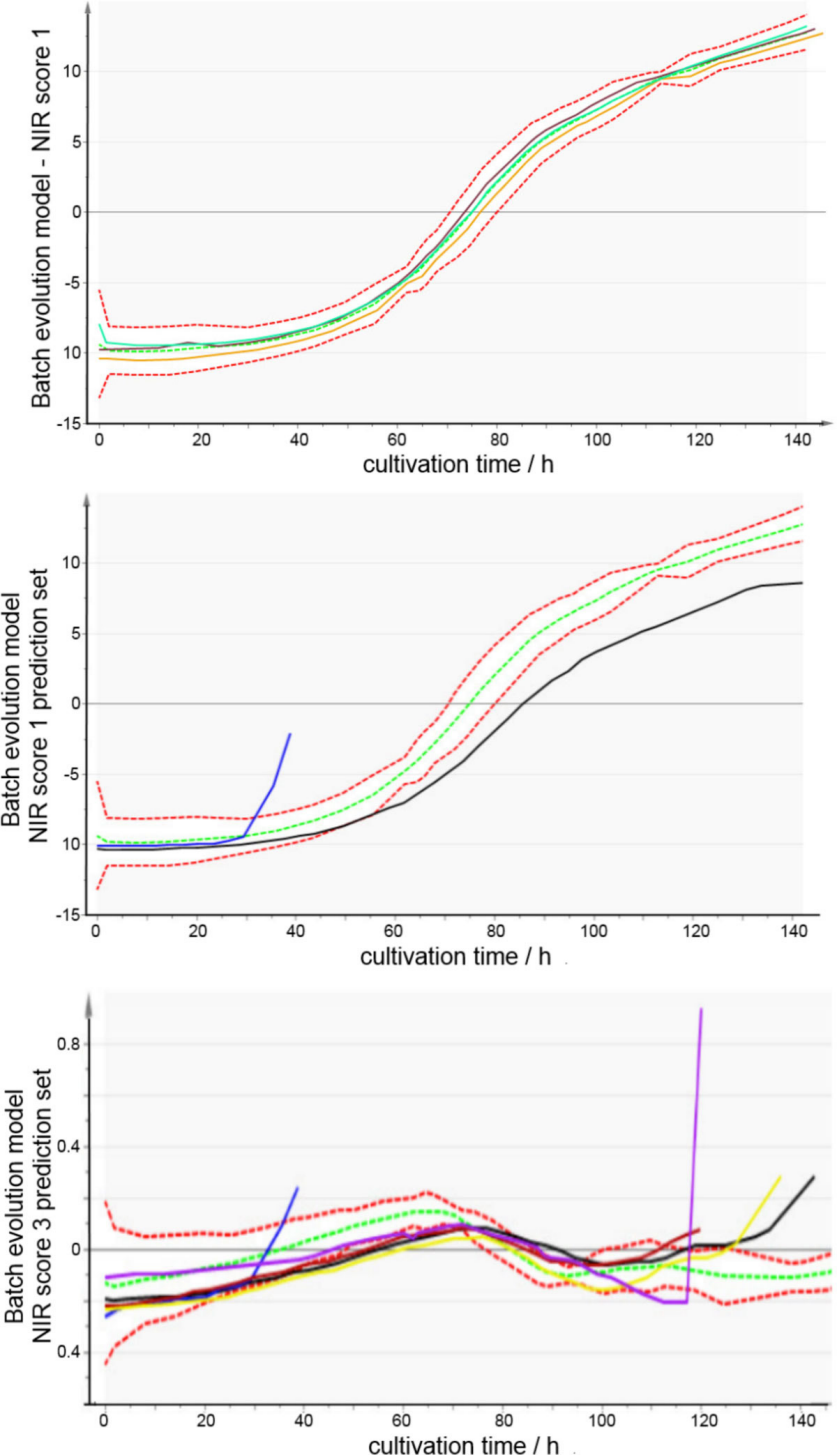
**Batch evolution models (BEM) based on NIR spectra**. **(Top): **Batch trajectories from three batches based on the first principal component of NIR spectra. The golden batch trajectory is shown in green (mean value of all contributing batches) and the process limits are shown in red (three times the standard deviation of the three contributing batches). **(Middle): **Compared to the BEM other batches show deviations which can be assigned to contaminations (blue line) or low cell growth rate (black line). **(Bottom**): Batch trajectories from three batches based on the third principal component of NIR spectra. Compared to the BEM other batches show deviations like contaminations (blue and violet line) or early glucose limitation which led to an early drop of viability (black, yellow and violet line).

Additional benefit is generated via MSPC of NIR data. Batch trajectories have been generated from major variances of the NIR spectra. The Score values have been used and plotted over time using SIMCA 13. Figure 1 (top) shows the BEM build for the first principal component of the NIR spectra. Three batches contribute to this model, which showed optimal cell growth. All batches show almost an identical profile which indicates a high batch-to-batch reproducibility, both in terms of process operation and spectra acquisition. The mean trajectory (green dashed line) is called golden batch and represent the profile of optimal performance. Moreover, process limits (red dashed lines) can be defined, which are calculated by three times the standard deviation of the batches involved in the model. Other batches can be compared to the model. As long as the trajectory of a new batch stays within the limits, it can be assigned as statistically identical to the golden batch. A relevant process deviation will be notified if the trajectory is outside of the limits. Significant process deviations are shown in Figure 1 (middle). The trajectory of batch 3 (blue line) surpasses the process limits after 30 h. The reason for this was a bacterial contamination during the process. In batch 2 (black line) a different aeration strategy was applied which resulted in a lower cell growth rate. In Figure 1 (bottom) a BEM based on the third principal component is shown. The model (dashed lines) is again generated from high performance batches as seen in the model above.

## Summary

The Ingold port adaption of a free beam NIR spectrometer is tailored for optimal bioprocess monitoring and control. The device shows an excellent signal to noise ratio dedicated to a large free aperture and therefore a large sample volume. This can be seen particularly in the batch trajectories which show a high reproducibility. The robust and compact design withstands rough process environments as well as SIP/CIP cycles.

Robust free beam NIR process analyzers are indispensable tools within the PAT/QbD framework for real-time process monitoring and control. They enable multiparametric, non-invasive measurements of analyte concentrations and process trajectories. Free beam NIR spectrometers are an ideal tool to define golden batches and process borders in the sense of QbD. Moreover, sophisticated data analysis both quantitative and MSPC yields directly to a far better process understanding. Information can be provided online in easy to interpret graphs which allow the operator to make fast and knowledge-based decisions. This finally leads to higher stability in process operation, better performance and less failed batches.
